# Extracellular vesicles in the pathogenesis and treatment of acute lung injury

**DOI:** 10.1186/s40779-022-00417-9

**Published:** 2022-11-01

**Authors:** Qian Hu, Shu Zhang, Yue Yang, Jia-Qi Yao, Wen-Fu Tang, Christopher J. Lyon, Tony Ye Hu, Mei-Hua Wan

**Affiliations:** 1 Department of Integrated Traditional Chinese and Western Medicine, West China Hospital of Sichuan University, Chengdu, 610041 China; 2 Department of Emergency Medicine, Emergency Medical Laboratory, West China Hospital of Sichuan University, Chengdu, 610041 China; 3 Center of Cellular and Molecular Diagnosis, Tulane University School of Medicine, 1430 Tulane Ave., New Orleans, LA 70112 USA; 4 Department of Biochemistry and Molecular Biology, Tulane University School of Medicine, 1430 Tulane Ave., New Orleans, LA 70112 USA; 5 West China Hospital (Airport) of Sichuan University, Chengdu, 610299 China

**Keywords:** Acute lung injury (ALI), Acute respiratory distress syndrome (ARDS), Extracellular vesicles (EVs), Pulmonary inflammation, Mesenchymal stem cells (MSCs)

## Abstract

Acute lung injury (ALI) and acute respiratory distress syndrome (ARDS) are common life-threatening lung diseases associated with acute and severe inflammation. Both have high mortality rates, and despite decades of research on clinical ALI/ARDS, there are no effective therapeutic strategies. Disruption of alveolar-capillary barrier integrity or activation of inflammatory responses leads to lung inflammation and injury. Recently, studies on the role of extracellular vesicles (EVs) in regulating normal and pathophysiologic cell activities, including inflammation and injury responses, have attracted attention. Injured and dysfunctional cells often secrete EVs into serum or bronchoalveolar lavage fluid with altered cargoes, which can be used to diagnose and predict the development of ALI/ARDS. EVs secreted by mesenchymal stem cells can also attenuate inflammatory reactions associated with cell dysfunction and injury to preserve or restore cell function, and thereby promote cell proliferation and tissue regeneration. This review focuses on the roles of EVs in the pathogenesis of pulmonary inflammation, particularly ALI/ARDS.

## Background

In 1994, the American-European Consensus Conference (AECC) defined that acute lung injury (ALI) is characterized by rapid-onset respiratory failure, with an oxygen partial pressure to inspired fraction (PaO_2_/FiO_2_) ratio of ≤ 300 mmHg (regardless of the positive end-expiratory pressure level), a chest radiograph showing bilateral infiltration, and pulmonary capillary wedge pressure ≤ 18 mmHg (no left atrial hypertension); acute respiratory distress syndrome (ARDS) is defined identically but with an even lower limiting value of < 200 mmHg for PaO_2_/FiO_2_ [1–4]. Although ALI/ARDS are “syndromes” caused by different injuries and conditions, their similar pathobiology of lung injuries, clinical manifestations, and specific targets of drug intervention, have resulted in ALI and ARDS being studied as one entity. ALI/ARDS cases are referred to as mild (PaO_2_/FiO_2_ ratio of 201 – 300 mmHg), moderate (PaO_2_/FiO_2_ ratio of 101 – 200 mmHg), and severe (PaO_2_/FiO_2_ ratio ≤ 100 mmHg) in the later Berlin Definition [5]. Both ALI and ARDS have substantial mortality, and current treatment still emphasizes symptomatic and supportive treatment after the occurrence of the disease, including mechanical ventilation, prophylactic or therapeutic application of antibiotics and restriction of fluid accumulation, as well as the treatment of initial injury or disease [2]. Even with treatment, ALI/ARDS usually leads to substantial respiratory failure and mortality, with in-hospital mortality rates up to 38 – 46% [6, 7]. Patients who survive also frequently suffer long-term physical, psychological, and/or cognitive dysfunction [1]. Thus, therapeutic approaches are urgently needed to prevent or control the pulmonary inflammatory cascade to reduce mortality and long-term morbidity by ALI/ARDS.

It was well known that alveolar cells maintain the stability of alveolar structure, and disruption of alveolar-capillary barrier integrity or activation of inflammatory responses cause dysfunctions in alveolar cells, lead to accumulation of proteinaceous edema and inflammatory cells in the alveolar space, and eventually progress to ALI/ARDS [6, 8]. The cells involved in the regulation of lung injury and their relevant mechanisms are different in lung injury models caused by different etiologies. For example, in the cell model of SARS-CoV-2 infection, a pathogenic stimulus that directly affects the lung epithelium activates an innate immune response to trigger acute lung inflammation [9, 10]. In indirect ALI/ARDS model (e.g., severe acute pancreatitis), lung microvascular endothelial cell injury caused by an extrapulmonary insult is the earliest cellular event and leads to interstitial edema [11]. Mounting evidence indicates that ALI/ARDS is influenced by gender. Women are more likely to develop ALI/ARDS after severe trauma than men, but men appear to have higher ALI/ARDS-associated mortality rates [12]. Mortality rate in men appears to correlate with testosterone level since male SARS-CoV-2 patients with lower total and free testosterone levels have poor prognoses [13]. This may be because that testosterone could enhance the responsiveness of the carotid body to hypoxia [14] and attenuate tissue inflammation, fibrosis, and hypoxia induced in response to a systemic pro-inflammatory syndrome [15]. However, unlike that in men, higher total and free testosterone levels are associated with more severe inflammatory states in women with SARS-CoV-2 pneumonia [16]. However, it is unclear if testosterone therapy would attenuate the development of ALI/ARDS or attenuate its pathogenesis in men, this approach would appear to be detrimental in women, where testosterone therapy is approved for only limited applications [17]. Better understanding of the mechanisms involved in different ALI/ARDS injury models is therefore crucial to determine effective means to prevent ALI/ARDS development, restore alveolar function, reduce lung inflammation, improve prognosis, and reduce mortality.

Extracellular vesicles (EVs) are lipid bilayer-enclosed extracellular particles that are released by all cell types under both normal and disease conditions [18]. EVs are classified into three major groups: apoptotic bodies, microvesicles (MVs), and exosomes – by the International Society of EVs based on their sizes, membrane composition, and biogenesis mechanism [19]. As intracellular messengers, EVs can transport proteins, nucleic acids, lipids, and mitochondrial material from their parent cells that can regulate the activity of recipient cells upon they are transferred by membrane fusion, paracrine signaling, phagocytosis, or receptor-mediated endocytosis [20, 21]. Moreover, EVs secreted by both pulmonary and extrapulmonary tissues are reported to regulate physiological and pathological activities in lung tissues by mediating alveolar cell crosstalk events in the lung microenvironment [22, 23]. For example, EVs derived from epithelial and endothelial cells regulate alveolar macrophage inflammation and function [24], while EVs secreted by resident alveolar macrophages can blunt inflammatory signaling in alveolar epithelial cells (AECs) [25]. EVs are therefore responsible for the exchange of information between alveolar cells, and since they carry specific factors that reflect the phenotype of their original cells, including the disease state, they represent promising candidates for diagnosing pathological lung injury events, predicting disease development and blocking the processes of ALI/ARDS.

Mesenchymal stem cells (MSCs), an adult stem cell subtype that has immunomodulatory functions, are reported to have the potential to repair damaged lung tissue and alleviate ALI/ARDS in various preclinical models. These beneficial effects are mediated by a variety of mechanisms, including reducing tissue inflammation and the permeability of the alveolar epithelial and endothelial cells, enhancing alveolar fluid clearance and macrophage phagocytic activity, and directly transferring mitochondria to lung cells [26]. Recently, increasing evidence indicates that MSC-mediated therapeutic effects primarily derive from paracrine mechanisms, regulated by cytokines and EVs. MSC-derived EVs (MSC-EVs) also attenuate inflammation, promote alveolar epithelium regeneration, repair microvascular permeability, and prevent fibrosis in different ALI models [27]. Further, MSC-EVs not only retain the biological functions of MSCs but are more stable and less prone to stimulating tumorigenesis. MSC-EVs have thus become a new, cell-free treatment strategy for ALI/ARDS [28]. However, it is controversial whether or not MSC-EVs can completely replace key MSC activities in the treatment of ALI/ARDS. For example, it has been reported that cytokines secreted by MSCs, but not MSC-EVs treatment, prevent thrombin-induced pulmonary vascular endothelial permeability [29]. A comprehensive exploration of the therapeutic effects and mechanisms of MSC-EVs in attenuating pulmonary inflammation and injury is needed to support their use in the clinical prevention and treatment of ALI/ARDS.

Thus, this review provides a comprehensive overview of the relationship between EVs and the various alveolar cells involved in mediating ALI/ARDS, with a special focus on their roles in pathogenesis and their potential applications as diagnostic biomarkers and therapeutic strategies for ALI/ARDS.

## Alveolar cells and their roles in ALI/ARDS

Alveoli are the main structural and functional units of pulmonary gas exchange, and multiple cell types contribute to the maintenance of alveolar integrity and homeostasis. These cells are important for sustaining alveolar function and preventing the colonization of alveoli by microbial pathogens [30]. Once alveolar dysfunction, for instance, the destruction of the pulmonary air-blood barrier and the activation of immune cell inflammatory responses could cause pulmonary inflammation and injury [31, 32]. The lung air-blood barrier, which includes the alveolar epithelial and vascular endothelial barriers, performs several vital functions by maintaining alveolar structure and the balance of alveolar fluid clearance and material exchange [33]. But under non-physiological stress conditions, mechanical transduction between AECs and vascular endothelial can regulate pathophysiological process [34]. AECs experience mechanical strain during normal breathing which results in different stresses at their apical and basolateral surfaces [35]. This stress regulates type II alveolar epithelial cells (AEC-IIs) proliferation, migration, survival and secretion [36–38], while type I alveolar epithelial cells (AEC-Is), which derive from AEC-IIs and do not exhibit proliferative capacity, respond to periodic stretching to regulate permeability of the epithelial cell junctions and can release ATP to promote AEC-II apoptosis, monocyte recruitment, and inflammation [39]. Vascular endothelial cells are also subject to cyclic stretching during respiration and are, for example, prone to injury during mechanical ventilation [40]. Similarly, non-physiological mechanical stresses encountered excess vascular stretch in response to pulmonary hypertension or loss of epithelial and endothelial elasticity due to interstitial fibrosis may also promote injury to these cell types. Here, we provide an overview of the function of alveolar cells in the context of alveolar maintenance in health and disease.

### Alveolar epithelium

The alveolar epithelium contains AEC-I and -II. Large and flat AEC-Is are primarily responsible for the structure of the alveolar wall as they cover approximately 90% of alveolar surface area, despite accounting for only 8% of the pulmonary cell population [30]. AEC-Is thus play key roles in gas exchange between alveoli and capillary blood and are also important in the regulation of pulmonary innate immune responses, cell proliferation, ion and water transport, and regulatory peptide metabolism [30]. In contrast, small and rectangular AEC-IIs account for about 16% of all pulmonary cells but cover only about 7% of alveolar surface area [30]. These cells are highly plastic and are able to trans-differentiate into AEC-Is [30] to restore alveolar structure and function after injury. AEC-IIs also secrete alveolar surfactants, regulate surfactant metabolism to maintain alveolar function, and express innate immune factors that regulate local inflammation [41]. Epithelial cell dysfunction is recognized as an important factor contributing to ALI/ARDS progression, as it can decrease surfactant production and destabilize alveolar barriers, thereby facilitating pulmonary fluid accumulation and the migration of inflammatory cells into alveolar space [42]. And ALI severity positively correlates with the extend of AEC apoptosis and autophagy, and enhanced autophagy of lung epithelium has been observed in mice with lipopolysaccharide (LPS)-, oleic acid-, and cecum ligation and puncture-induced ALI [41, 42]. Thus, inhibition of autophagy and apoptosis of the lung epithelium may be an effective strategy to reduce lung inflammation. Besides, regulation of molecular signaling in AECs could also be used to inhibit pulmonary inflammation leading to tissue injury. For example, one study has suggested that transfection of AECs with a miRNA mimic could be used to inhibit pulmonary inflammation leading to ALI/ARDS. This study reported that serum of patients with ARDS and an AEC model of ARDS were enriched with overexpressed miR-155-5p, which interact with complementary sequence in three interleukin receptor transcripts to down-regulate their expression and the ability of these receptors to propagate pro-inflammatory signal cascades [43]. Further, transfection of LPS-induced AECs with a miR-155-5p mimic significantly inhibited inflammation-induced injury [43], suggesting that interventions targeting alveolar epithelium signaling pathways could serve to reduce lung inflammation and injury induced by pro-inflammatory cytokine exposure.

### Pulmonary microvascular endothelium

The vascular endothelium lines the entire circulatory system and maintains the integrity of vessels that regulate the passage of cells and other materials [44], and the pulmonary microvascular endothelium and AECs form a blood-air barrier that serves a similar function [42]. However, unlike pulmonary artery and venous endothelial cells, the pulmonary microvasculature endothelium is a major metabolic structure that forms a highly impermeable barrier that normally prevents the free passage of water, solutes, pathogens, and inflammatory cells between the blood and interstitium [45, 46]. In addition to this barrier activity, pulmonary microvasculature endothelium is involved in the clearance of several important regulatory factors from the circulation, including angiotensin I, bradykinin and endothelin [45, 46]. It also produces vasoconstrictor and vasodilator substances (angiotensin II), nitric oxide and prostacyclin to regulate coagulation, hemostasis and cell proliferation [45, 46], and may regulate the innate immune response by secreting inflammatory mediators and recruiting neutrophils [44, 46]. Abnormal pulmonary microvasculature endothelial cell proliferation, apoptosis, or necrosis, or the weakening of endothelial cell connections leads to endothelial dysfunction and a loss of their barrier integrity. This allows pro-inflammatory mediators [e.g., interleukin-18 (IL-18) and tumor necrosis factor-α (TNF-α)], inflammatory factors [e.g., reactive oxygen species (ROS)] and inflammatory cells (e.g., neutrophils) to enter the alveolar space and up-regulate endothelial cell adhesion molecules [e.g., intercellular cell adhesion molecule-1 (ICAM-1) and -2 (ICAM-2)], which further attract inflammatory cells (including neutrophils and monocytes/macrophages). This amplification of the inflammatory response ultimately promotes the development of ALI/ARDS [44]. Thus, inhibiting endothelial cell apoptosis, promoting endothelial cell regeneration, and restoring intercellular endothelial cell junctions could prevent or reduce pulmonary inflammation. For example, the mitochondria-targeted antioxidant MitoQ can reduce lung inflammation by inhibiting endothelial cell apoptosis and preventing the rupture of intercellular junctions between endothelial cells [47]. Notably, pulmonary endothelial dysfunction can also induce other chronic lung diseases, including chronic obstructive pulmonary disease (COPD), pulmonary hypertension, and obstructive sleep apnea [48]. Restoring pulmonary microvasculature endothelial cell barrier function is therefore an important strategy for preventing and treating ALI.

### Macrophages

In addition to epithelial and endothelial cells, some immune cells are involved in maintaining the functional stability of alveoli, and over-activated immune cells may be responsible for the development of ALI/ARDS. Macrophages are the main immune cell population involved in regulating lung inflammation, and produce pro-inflammatory factors (e.g., TNF-α, IL-1, IL-9 and IL-8), inflammatory mediators (e.g., elastin, cathepsins, collagenases and gelatinases), cytokines, and chemokines that can damage or impair the function of endothelial and epithelial cells [49]. Pulmonary macrophage populations can be polarized to adopt pro-inflammatory “M1” and the anti-inflammatory “M2” phenotypes. Under inflammatory conditions, alveolar macrophages are polarized to an M1 phenotype and peripheral blood monocytes are recruited into the alveolar lumen where they primarily differentiate into M1 macrophages. The differentiation of invading monocytes to M1 or M2 phenotypes is regulated by factors present in the microenvironment. For example, monocytes undergo differentiation and polarization to an M1 phenotype in response to exposure to a large number of soluble pro-inflammatory factors and via the activation of specific signaling pathways that regulate nuclear factor kappa-B, mitogen-activated protein kinase, and NLR family pyrin domain containing 3 (NLRP3) [50–53]. M1 macrophages secrete factors, such as macrophage inflammatory protein-2 (MIP-2) and IL-8, that can recruit monocytes and neutrophils and promote the development of pulmonary inflammation that eventually leads to ALI/ARDS [54]. Conversely, M2 macrophages secrete anti-inflammatory and pro-angiogenic factors and phagocytose apoptotic cells to promote tissue remodeling [53, 55]. Further, in an LPS-induced mouse ALI model, alveolar macrophages were reported to release pyroptotic bodies containing damage-associated molecular patterns that could mediate pulmonary inflammation by promoting epithelial cell activation, inducing vascular leakage, and recruiting neutrophils [56]. Therefore, inhibiting this pro-inflammatory macrophages response by promoting macrophage M1 to M2 polarization [57], inhibiting NLRP3 inflammasome activation and macrophage apoptosis [58], and inducing macrophage mitophagy to prevent pyroptosis [59] may be effective strategies to reduce lung inflammation. It has also recently been reported that androgen exposure can induce macrophages to adopt pro- or anti-inflammatory phenotypes. For example, dihydroxy testosterone exposure can promote M1 macrophage polarization in vitro, while cypionate testosterone treatment can induce an androgen-dependent increase in M2 macrophage polarization in a female mouse infection model [60]. Evidence also suggests that androgens can regulate oxytocin levels, which have been proposed to attenuate cytokine storms, lymphocyte deficiency, thrombosis, ALI/ARDS and organ failure, leading to its proposed use as a treatment for COVID-19 pathogenesis [61]. Oxytocin has also been reported to reduce the M1/M2 macrophage ratio and TNF-α expression in M1 macrophage [62], mediate anti-inflammatory and antioxidant activity during sepsis-induced ALI [63], and exhibit estrogen regulation [64]. Taken together, these results suggest that sex hormones may exert differential effects on inflammatory responses, including ALI/ARDS, through complex regulatory effects on macrophages, and potentially other cell types.

### Neutrophils

Neutrophils are the first cells recruited across the endothelial and epithelial cell barriers to the sites of inflammation in the alveolus during pulmonary inflammation [65]. Thus, excessive neutrophil activation and transmigration are considered hallmark events in the progression of ALI and ARDS [65, 66]. Activated neutrophils would release cytotoxic and immune cell-activating agents, such as granule proteins, cationic polypeptides, ROS, and serine proteases, or form neutrophil extracellular traps (NETs) to capture pathogens [65, 67]. However, excessive responses, including NET formation, will promote pro-inflammatory macrophage phenotypes and induce pulmonary epithelial injury, which cause severe lung inflammation [66, 67]. For example, in a mouse ALI model of influenza-induced pneumonia, macrophage-depleted mice exhibited similar pathological features of ALI/ARDS as influenza-infected wild-type mice, including diffuse alveolar injury, pulmonary edema, hemorrhage and hypoxemia. Conversely, the lungs of neutrophil-depleted influenza virus-infected mice revealed only slight pathological changes [68]. This indicates that neutrophils play a key role in the development of ALI/ARDS, and neutrophils damage the lung epithelium and endothelium, impair their barrier functions, and trigger lung inflammation by forming NETs in this influenza-induced pneumonia mice [68]. Similarly, in an ALI mouse model induced by cutaneous chemical burns, phenylarsine oxide chemical burns activated NETs formation, which led to ALI via the destruction of pulmonary microvascular endothelial cell barrier integrity resulting from NET-induced cell death [69]. In addition to stimulating lung inflammation by disrupting the pulmonary endothelial cell barrier, NETs also promote the ARDS by regulating macrophage polarization. NET levels in patients with ARDS positively correlate with M1 macrophage polarization, and NET inhibitors induce significant down-regulation of M1 macrophage markers (e.g., inducible nitric oxide synthase) and up-regulation of macrophage M2 markers (e.g., CD206 and Arg1) [67].

However, acute respiratory failure with poor prognosis is associated with low systemic neutrophil level (neutropenia) after chemotherapy [70], and that neutrophils can attenuate the progression of lung injury [71]. In the early stages of lung inflammation, the neutrophils driven over-activation of the immune response can damage lung tissues. However, at later stages after the lung injury, neutrophils can promote lung tissue repair by devouring cell debris, promoting tissue neovascularization and secreting lipolytic mediators that relieve lung inflammation [72]. Thus, the role of neutrophils in pulmonary inflammation is complex and interventions that target neutrophil activities in ALI/ARDS must account for this dual role of neutrophils and the disease stage at intervention.

**Fig. 1:**
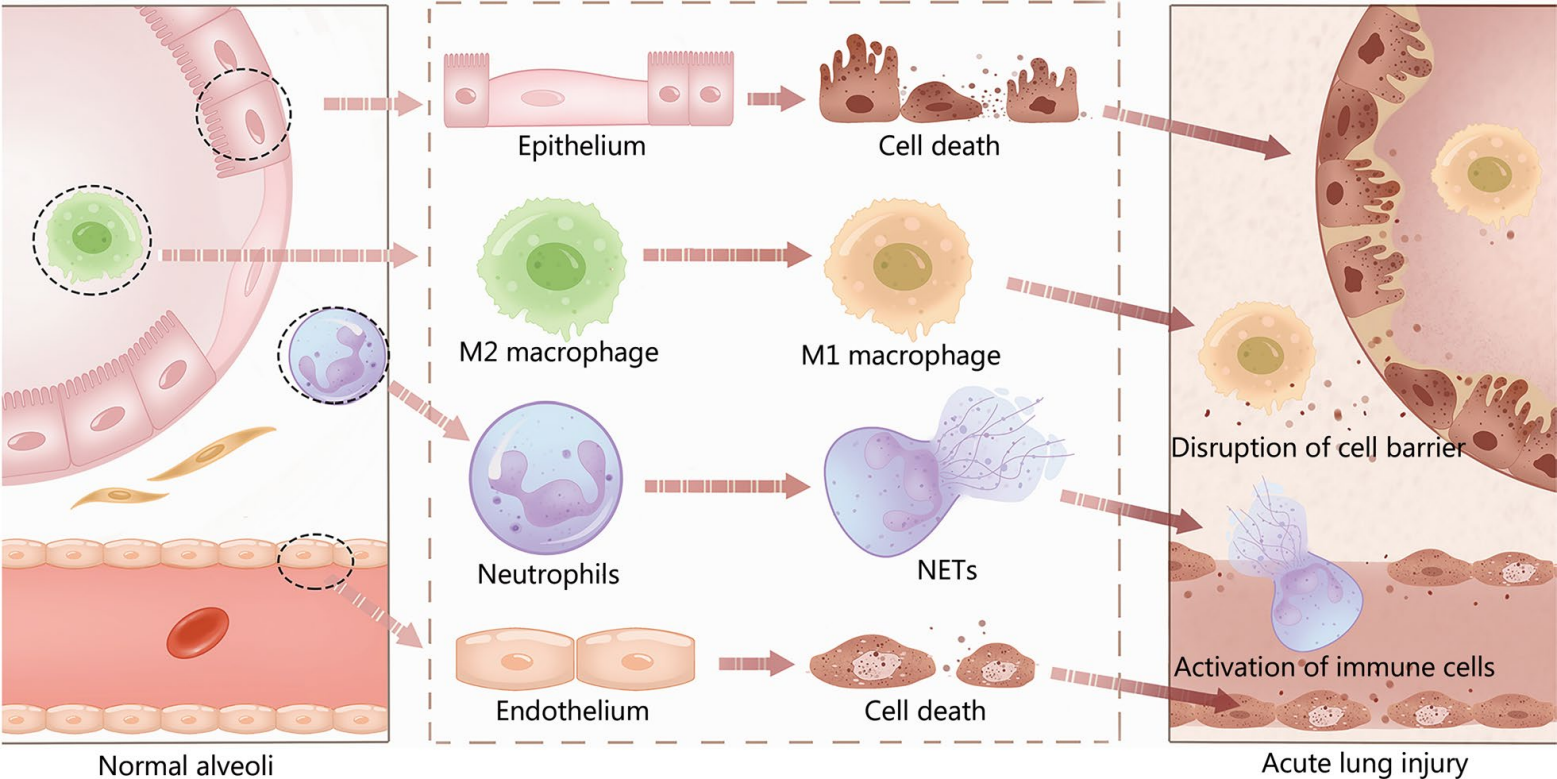
Alveolar cells in normal alveoli and acute lung injury. A variety of alveolar cell types, including epithelial cells, endothelial cells, alveolar macrophages and neutrophils, maintain alveolar functional integrity and homeostasis. The disruption of endothelial-epithelial barrier, the pro-inflammatory polarization of alveolar macrophage, and the formation of neutrophil extracellular traps (NETs) induce acute lung injury

The disruption of the endothelial-epithelial barrier and subsequent invasion and/or activation of pro-inflammatory cells (macrophages and neutrophils) play a critical role in ALI/ARDS disease (Fig. 1). However, the underlying mechanisms which alveolar cells employ to cooperatively promote the development of ALI/ARDS are still unclear.

## Role of EVs in regulating alveolar cells in ALI/ARDS

Since endothelial-epithelial dysfunction/disruption and immune cell recruitment and activation in lung tissue are the pathological basis of ALI/ARDS, a critical question is what factors or molecules regulate these events? EVs are actively involved in the functional homeostasis of pulmonary cells; however, under pathological conditions EVs secreted by various cell types in the lung microenvironment can carry cargoes that regulate pathological processes via a variety of pathways [18, 21]. For example, EV-mediated intercellular communication between cells in respiratory tissue through miRNA transfer influences the development and progression of multiple respiratory diseases, including COPD, asthma, ALI and pulmonary arterial hypertension [73]. In this section, we will discuss how EVs regulate alveolar cell phenotypes to induce ALI/ARDS.

### Effects of EVs on the alveolar epithelium

The airway epithelium is the first line of defense against the external environment [74], but under pathological conditions various cell types in the lung microenvironment secrete EVs that dysregulate airway epithelium processes [18]. For example, EVs from patients with septic shock have been found to encapsulate miRNAs and mRNAs related to pathogenic pathways that participate in inflammatory and oxidative stress responses, and regulate the cell cycle of recipient cells [75]. A separate study found that elevated miR-1298-5p in exosomes of patients with sepsis-related ALI invoked lung inflammation by inhibiting proliferation of human bronchial epithelial cells (BEAS-2B) and inducing epithelium permeability [74].

EV-based crosstalk between stressed immune and lung epithelial cells also plays a role in promoting the development of lung inflammation. For instance, following stress, infection, or hyperoxia, lung cells abundantly release EVs that contain pathological cargoes into airway surface liquid (ASL) to disrupt the balanced crosstalk between the lung epithelium and immune cells, leading to pulmonary inflammation and injury [76]. In addition to their role in inducing acute pulmonary inflammatory reactions, EVs also shuttle cargoes that modulate cell signaling and injury responses in chronic pulmonary inflammatory diseases, including COPD and asthma. For example, T lymphocyte-derived EVs from COPD patients can lead to injury and inflammation of airway epithelial cells by reducing cell viability, promoting inflammatory cytokine production, and decreasing anti-inflammatory cytokine levels [77]. In asthma pathogenesis, nuclear polymorphisms in suppression of cytokine signaling 1 (SOCS1) and SOCS3 are associated with allergic inflammation, and reduced levels of SOCS3 were found in bronchoalveolar lavage fluid (BALF) samples of asthmatic as well as allergen- and cigarette smoke-challenged mice [25]. Alveolar macrophage-derived EVs containing SOCS1 and SOCS3 can inhibit asthma pathogenesis when taken up by AECs [25, 78]. Thus, EVs that carry factors with pro- or anti-inflammatory activity can act on the airway and alveolar epithelia to induce or suppress pathological reactions that participate in ALI/ARDS.

### Effects of EVs on the vascular endothelium

Pulmonary microvascular endothelial cells form the barrier between the blood and pulmonary interstitium, and play important roles in regulating pulmonary vascular tension, maintaining vasomotor balance, and controlling adhesion and metastasis of inflammatory cells [79]. When this endothelial cell barrier is damaged, permeability increases and protein-rich exudate and immune cells leak into the alveolar space, resulting in ALI/ARDS [80]. Circulating EVs carrying disease-associated molecules can disrupt the endothelium and activate downstream signaling pathways that regulate endothelial cell inflammation, oxidative stress, and apoptosis, resulting in serious leakage of the pulmonary vasculature, interstitial edema, and the initiation and progression of lung injury [81, 82]. Further, pro-inflammatory activation of pulmonary microvascular endothelial cells induces the expression of cell adhesion molecules that allow recruited primed neutrophils to form firm neutrophil-endothelial cell adhesions that promote ALI/ARDS development [83].

ALI/ARDS is the main cause of death in patients with sepsis, and EV miRNA cargoes play key roles in endothelial cell dysfunction during sepsis. For example, miR-1-3p expression is significantly increased in EVs isolated from sepsis patients and from cecal ligation and puncture sepsis rat models. This elevated miR-1-3p expression can inhibit endothelial cell proliferation and increase endothelial contraction, membrane injury, monolayer permeability, and apoptosis by inhibiting stress-associated endoplasmic reticulum protein 1 (SERP1) expression [82]. Similarly, another study reported that miR-210-3p-enriched plasma EVs from sepsis patients can enhance multiple phenotypes associated with sepsis-induced ALI in cultured cells, including macrophage inflammation, bronchial epithelial cell apoptosis, and pulmonary microvascular endothelial permeability by regulating autophagy and inflammation [84]. In addition to transporting miRNAs that can damage the lung endothelial cells, circulating EVs also carry proteins that play vital roles in destroying the endothelial barrier. For example, plasma exosomes of patients with burn injury are enriched with the S100 calcium binding protein A9 (S100A9), which inhibits the expression of zonula occludens-1 (ZO-1) and occludin, and activates the p38 MAPK signaling pathway in human pulmonary microvascular endothelial cells (HPMECs), ultimately leading to the disruption of the tight junctions and endothelial barrier [81]. Similarly, plasma EVs of hyperoxia-exposed rats revealed increased levels of surfactant protein C and GSDMD-p30, and their injection into new-born rats induced inflammatory injury and cell death in pulmonary vascular endothelial cells [85].

EVs also transfer some proteins which are beneficial to alleviate endothelial cell-mediated lung injury. Expression of syndecan-1 in endothelial cells has the potential to protect barrier function and suppress inflammatory responses. Further, EVs loaded with syndecan-1 can mitigate the expression of pro-inflammatory cytokines, decrease stress fiber formation, and improve monolayer hyper-permeability in mouse pulmonary microvascular endothelial cells after LPS stimulation [86]. EV protein and miRNA cargoes can disrupt the endothelial barrier by increasing the expression of pro-inflammatory adhesion molecules and by modulating the activity of adherent junctions, tight junctions, and cytoskeletal proteins [87]. Also, regulating the levels of key proteins or miRNAs in circulating EVs can restore tight junctions between endothelial cells and has the potential to be a new treatment for reducing lung inflammation caused by endothelial cell injury.

### Effects of EVs on alveolar macrophages

ALI/ARDS is characterized by the activation of alveolar macrophages, which are located in the alveolus and are in direct contact with external pathogens. These macrophages participate in regulating lung inflammation and maintaining airway immune balance by secreting cytokines and chemokines and by phagocytosing pathogens and cell debris. Cytokines (e.g., Toll-like receptors) and other inflammatory factors (e.g., TNF-α, IL-6 and IL-1β) can induce ALI/ARDS through actions on alveolar macrophages and other cell types, as have been extensively reviewed in the literature [53]. Recent studies, however, now indicate that plasma EVs can also trigger inflammatory responses leading to ALI following their uptake by alveolar macrophages [88, 89]. And in a mouse model of sepsis-related ALI, two EV-based mechanisms were reported to participate in pulmonary inflammation by targeting macrophage SH2 domain-containing inositol phosphatase 1 (SHIP1) and SOCS1 expression. Serum EVs were found to promote macrophage proliferation by transferring miR-55, while EVs secreted by activated neutrophils induced pro-inflammatory M1 macrophage polarization and apoptosis by transferring miR-30d-5p [90, 91]. Thus, the polarization of alveolar macrophages in the progress of ALI/ARDS is partly attributed to the role of EVs.

EVs in ASL can directly contact alveolar macrophages, and EV-mediated macrophage pro-inflammatory activation is involved in promoting ALI. One study found that exosomes present in ASL samples from a rat model of cecal ligation and puncture-mediated septic lung injury were enriched with miR-92a-3p, which could induce macrophage-related inflammation [92]. Pretreatment with an exosome release inhibitor reduced exosome levels in the ASL and reduced pulmonary inflammation, thereby improving patients’ survival [92]. Similarly, BALF samples obtained from a hyperoxia-related oxidative stress mouse model of lung injury revealed elevated levels of EV-enriched with miR-320a and miR-221 [19]. Control mice treated with EVs isolated from the BALF of mice exposed to hyperoxic stress vs. normal oxygen by inhalation exposure revealed a differential macrophage-mediated pro-inflammatory response [19]. Taken together, these experiments demonstrate that EVs found in ASL or BALF can activate the inflammatory states in alveolar macrophages, leading to ALI.

However, multiple cell types secrete EVs into the airways or alveolus, and the identity of the cell type(s) that secrete the EVs that are primarily responsible for alveolar macrophage activation leading to ALI is debatable. In a *Pseudomonas aeruginosa-*induced mouse pneumonia model, a highly miRNA-enriched EVs population in BALF that acted on alveolar macrophages to activate inflammasomes and promote lung inflammation was found primarily to be secreted by AEC-Is [93]. And lung EVs released by epithelial cells were largely responsible for the activation of alveolar macrophages by transferring caspase-3 and activating the ROCK1 pathway [94]. This is inconsistent with the conclusion that the EVs in ASL primarily originate from AEC-Is during aseptic infection, while EVs present in ASL following pulmonary infection predominantly derive from alveolar macrophages [95]. EVs produced by alveolar macrophages in the latter case are likely to cause positive feedback responses, as the uptake of EVs secreted by activated macrophages can promote the expression of pro-inflammatory cytokines in recipient macrophages [96]. Further, another study reported that CD74^+^ EVs secreted by AEC-IIs had pro-inflammatory and anti-fibrotic effects on alveolar macrophages, while CD31^+^ EVs secreted by vascular endothelial cells had anti-inflammatory and profibrotic effects on alveolar macrophages [24]. However, while the first finding is consistent with other literature, the second is controversial, since EVs released by inflamed endothelial cells are enriched in numerous inflammatory makers, chemokines and cytokines that establish an endothelial cell-macrophage crosstalk to promote macrophage polarization towards pro-inflammatory phenotypes [97]. Conversely, EVs secreted by oxidized low-density lipoprotein (OXLDL)-treated endothelial cells express MALAT1, a protein that is capable of promoting anti-inflammatory macrophage polarization in cardiovascular disease [98]. Thus, more research is needed to determine the effects of endothelial cell-derived EVs on alveolar macrophages. We can conclude, at present, that both disease-related EVs and alveolar function-related EVs in peripheral circulation and the alveolus or airway can regulate ALI/ARDS progression by activating alveolar macrophages.

### Effects of EVs on neutrophils

Neutrophil infiltration is closely related to the severity of lung injury and is regulated by a complex network of chemokines released by multiple cell types [65]. However, few studies have reported on the interactions between EVs and neutrophils during ALI/ARDS progression. This section will discuss how EVs act on neutrophils to regulate lung inflammation.

Introducing bacterial membrane vesicles (prokaryotic EV analogs) into the nasal cavities of healthy mice stimulates lung inflammation by increasing inflammatory cytokines (e.g., IL-8, IL-6, ICAM-1, pro-IL-1β, and TNF-α) and neutrophil levels, in a process where cytokines positively increase neutrophil lung infiltration and both cytokines and neutrophil levels act to promote ALI/ARDS progression [99]. This study failed to answer two key questions, however: 1) is neutrophil migration to the lungs mediated by the effects of bacterial-derived EVs on alveolar cells or via the release of inflammatory cytokines; and 2) do EVs expressed surface proteins induce neutrophils infiltration into lung tissue?

One study found that thrombin-induced platelet EVs stimulated transfusion-related ALI by direct actions on neutrophils to induce the formation of NETs [100]. Similarly, other studies have also shown that neutrophils can also release EVs to promote lung inflammation. For example, the streptococcal toxin pneumolysin can promote NET formation in human and mouse lung tissue and EV secretion by neutrophils, which can activate platelets to synergistically cause ALI/ARDS [101]. This EV-based platelet-neutrophil crosstalk was also observed in a mouse model of acute chest syndrome due to pulmonary vascular occlusion. Platelets can secrete EVs carrying IL-1β and caspase-1 to attract neutrophils and form platelet-neutrophil aggregates that will cause blood flow disorders in the pulmonary arterioles of mice with sickle cell disease and ALI/ARDS [102]. Attenuation of this EV-based platelet-neutrophil crosstalk pathway may thus be an effective therapeutic strategy for the prevention of acute chest syndrome. EVs secreted by neutrophils also share characteristics of their parental cells. For example, neutrophil-derived EVs present in the lungs of COPD patients can cause COPD-like disease in mice via a neutrophil elastase-dependent mechanism, which appears to destroy lung extracellular matrix and alveolar structure to cause lung inflammation [103]. Thus, EVs appear to stimulate the development of ALI/ARDS by acting on neutrophils to induce the formation of NETs and promoting the secretion of EVs that activate platelets.

**Fig. 2:**
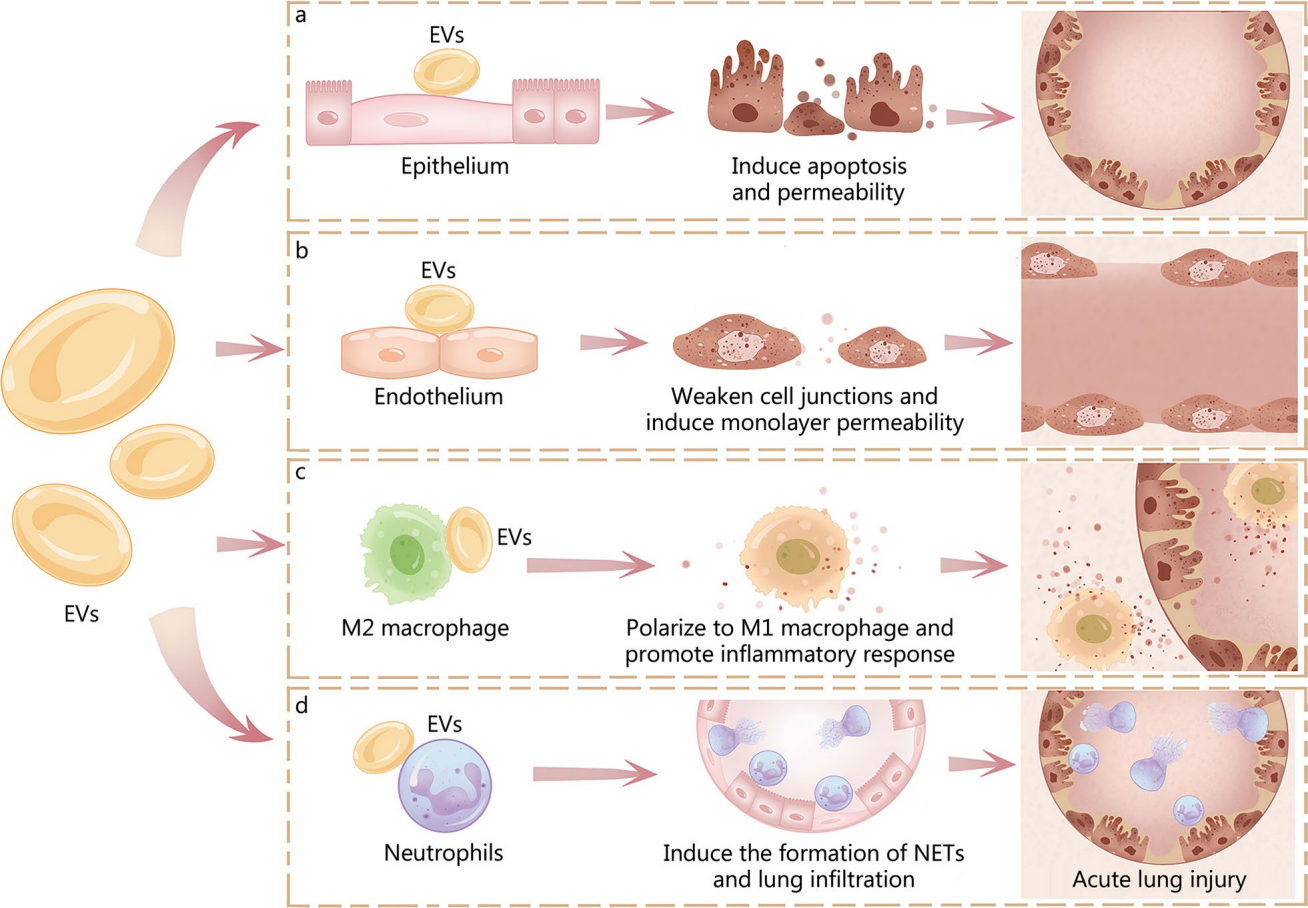
Pro-inflammatory EVs can promote the development of ALI/ARDS by regulating alveolar cell phenotype. **a** EVs induce lung epithelial cell apoptosis and permeability. **b** EVs weaken the pulmonary vascular endothelial cell junctions and barrier. **c** EVs promote M1 alveolar macrophage polarization. **d** EVs induce the formation of neutrophil extracellular traps (NETs) and neutrophil infiltration in lung. All these effects promote the development of ALI/ARDS. EVs extracellular vesicles, ALI/ARDS acute lung injury/acute respiratory distress syndrome

From the above studies, it is clear that EVs can regulate pulmonary inflammatory injury events by altering the structure and/or function of pulmonary epithelial cells and endothelial cells, the polarization state of pulmonary macrophages, the neutrophil migration and NETs formation (Fig. 2, Table 1).

**Table 1:** EVs participate in ALI/ARDS by regulating alveolar cells

EVs source	Molecules	Target cells	Effect	References
Human epithelial cell-EVs	miR-320a and miR-221	Macrophages	Activated macrophage and facilitated the recruitment of immunomodulatory cells	[19]
Vascular endothelial cell-EVs	CD31^+^ EVs	Alveolar macrophages	Anti-inflammatory and profibrotic effects	[24]
Type II alveolar epithelial cell-EVs	CD74^+^ EVs	Alveolar macrophages	Pro-inflammatory and anti-fibrotic effects	[24]
Alveolar macrophages-EVs	SOCS1 and SOCS3	Alveolar epithelial cells	Suppressed allergic inflammation	[25, 78]
Serum-exosomes	miR-1298-5p	BEAS-2B cells	Evoked inflammation, inhibited cell proliferation, and induced cell permeability	[74]
T lymphocyte-derived EVs	Unknown	Airway epithelial cells	Reduced cell viability, promoted inflammatory cytokine production, and decreased anti-inflammatory cytokine levels	[77]
Plasma-exosomes	S100A9 protein	HPMECs	Inhibited ZO-1 and occludin, and activated p38 MAPK signaling pathway/disruption of the tight junctions and endothelial barrier	[81]
Circulating blood-exosomes	miR-1-3p	HUVECs	Inhibited cell proliferation, promoted apoptosis and cytoskeleton contraction, increased monolayer endothelial cell permeability and membrane injury	[82]
Plasma-exosomes	miR-210-3p	THP-1 macrophage; BEAS-2B cells; HLMVECs	Enhanced THP-1 macrophage inflammation, BEAS-2B cell apoptosis, and HLMVEC permeability by regulating autophagy and inflammation activation	[84]
Plasma-EVs	Surfactant protein C and GSDMD-p30	Pulmonary vascular endothelial cells	Induced inflammatory injury and cell death	[85]
MPMVEC-exosomes	Syndecan-1 protein	Lung tissues	Attenuated ALI and inhibited inflammation	[86]
Peripheral serum-exosomes	miR-155	Alveolar macrophages	Reduced SHIP1 and SOCS1, promoted macrophage proliferation and inflammation	[90]
Activated neutrophils-derived EVs	miR-30d-5p	Alveolar macrophages	Induced polarization and apoptosis of M1 macrophages	[91]
Alveolar epithelial cells-exosomes	miR-92a-3p	Alveolar macrophages	Induced pulmonary inflammation	[92]
Alveolar epithelial cells-exosomes	miRNA	Alveolar macrophages	Activated inflammasomes and promoted lung inflammation	[93]
Lung epithelial cell-EVs	Caspase-3	Alveolar macrophages	Activated macrophages via the ROCK1 pathway	[94]
Dust bacteria	Unknown	Airway epithelial cells	Increased the inflammatory cytokines and neutrophil infiltration	[99]
Platelet	Unknown	Neutrophils	Mediated NETs formation	[100]
Neutrophils	Unknown	Platelet	Promoted platelet activation	[101]
Platelet	IL-1β and caspase-1	Neutrophils	Attracted platelet-neutrophile aggregates	[102]
Neutrophils	Neutrophil elastase and α−1 antitrypsin-resistance	Lung tissues	Destroyed lung extracellular matrix and alveolar structure	[103]

*ALI* acute lung injury, *ARDS* acute respiratory distress syndrome, *BEAS-2B* human bronchial epithelial cells, *EVs* extracellular vesicles, *HLMVECs* human lung microvascular endothelial cells, *HPMECs* human pulmonary microvascular endothelial cells, *HUVECs* human umbilical vein endothelial cells, *IL-1β* interleukin-1β, *MPMVECs* mouse pulmonary microvascular endothelial cells, *NETs* neutrophil extracellular traps, *SHIP1* SH2 domain-containing inositol phosphatese1, *SOCS1* suppressor of cytokine signaling 1, *SOCS3* suppressor of cytokine signaling 3, *S100A9* S100 calcium binding protein A9, *ZO-1* zonula occludens-1

## Role of EVs in the diagnosis of ALI/ARDS

EVs derived from pulmonary tissue and cells carry factors associated with lung injury. Under physiological conditions, the EVs secreted by lung cells have protective effects that can attenuate stress signals and contribute to the maintenance of lung homeostasis [104]. However, when lung cells are subjected to external stresses, such as hypoxia, hyperoxia, inflammatory factors or pathogens, they may alter the amount or composition of the EVs they secrete; these changes can serve as characteristic signals of lung injury that can be detected in the circulation. For example, the number of EVs dramatically increase during many cell stress responses. Specific stimuli, including hypoxia and endothelial cell activation, can also induce selective changes in EV RNA and protein content that reflect the altered phenotype of lung cells, while exposure to other stimuli (e.g., high glucose) may have modest or non-obvious effects on EV protein and RNA composition [105]. Thus, identifying changes in EV abundance and composition in the context of pulmonary disease may provide new clues for the etiological diagnosis of ALI/ARDS, or target therapies to inhibit the progression of the disease.

### EVs in serum as a diagnostic marker of ALI/ARDS

Ideally, biomarkers of lung injury and disease should be detectable in samples that can be obtained by low-invasive procedures (e.g., urine, blood, BALF, or tracheal aspirates) to facilitate diagnosis. To this end, changes detected in EVs present in the blood and BALF of ALI/ARDS patients have attracted great attention. For example, an observational clinical study found that septicemia patients with high levels of miR-34a and low levels of miR-15a and miR-27a in their plasma exosomes were more prone to shock, and that shock could be predicted by the combined level of these three miRNAs [106]. Similarly, in patients with severe community-acquired pneumonia, the combined EV expression of several miRNAs, including miR-126, miR-27a, miR-146a and miR-155, was predictive of ARDS, and miR-126 predicted 28-day mortality [107]. Therefore, serum EVs can be used not only as a biomarker of ALI/ARDS, but also to predict the disease development, which may permit interventions that can arrest disease development or attenuate its progression. In addition, some molecules that serve as disease biomarkers may also function as therapeutic targets if they contribute to the injury responses that lead to ALI/ARDS. For example, Cao et al*.* [108] reported that EV miR-145 level was significantly decreased in blood samples of an LPS-induced mouse model of ALI, similar to the results from sepsis patients; and intravenous injection of a miR-145 agomir into this mouse model, before or after LPS treatment, could attenuate lung inflammation and injury by attenuating TGFBR2 signaling. Thus, exploring the transcriptome of serum EVs of patients with different diseases and identifying transcripts related to development of diseases has great potential to improve disease diagnosis, predict disease prognosis, and identify therapeutic targets for new interventions. However, serum EV biomarkers may not be sufficiently specific for ALI/ARDS diagnosis since they are secreted by multiple cell types during tissue injury, particularly in ALI/ARDS cases that arise from excessive systemic inflammatory responses, such as sepsis-induced ALI.

### EVs in BALF as diagnostic markers of ALI/ARDS

EVs in BALF samples of ALI/ARDS patients are reported to mediate inflammatory responses and alveolar cell crosstalk during ALI pathogenesis, and thus can directly participate in the progression of ALI/ARDS [109]. For example, both alveolar macrophages and lung epithelial cells secret EVs into the alveolus following LPS exposure, but by one hour post-LPS stimulation alveolar macrophages are the dominant source of pro-inflammatory EVs [110]. These EVs, which are highly enriched for TNF but contain minimal IL-1β and IL-6, can induce lung epithelial cells to up-regulate expression of ICAM-1 and release keratinocyte-derived cytokine (CXCL1), which attracts more intensive neutrophil infiltration and induces lung inflammation [110]. Moreover, these EVs have also been proposed to induce neutrophils to release inflammatory cytokine IL-10, which may promote alveolar macrophages to polarize to a pro-fibrotic M2c macrophage phenotype and generate crosstalk between alveolar macrophages and neutrophils to induce lung damage [109]. BALF EVs are thus derived from a diverse population of cells, as pulmonary epithelial cells, neutrophils and alveolar macrophages can secrete EVs, and EV-mediated interactions among these cells can synergistically promote ALI/ARDS development. In addition, changes in specific EV markers in BALF samples may be used as diagnostic tools in lung injury, because EVs produced by lung cells during microbial infections may also contain factors characteristic of specific pathogens. For example, *Streptococcus pneumonia* infections can cause ALI. In this process, pneumococcal toxin pneumolysin stimulates lung epithelial cells to release EVs containing mitochondrial material, which can be transferred to neutrophils to suppress their ability to produce ROS involved in the innate immune response [111]. Thus, EV biomarkers associated with lung injury may vary according to the type of pathologic stimuli and the types of affected cells from which they are secreted. Specific cytokines in EVs (e.g., TNF and IL-10) in BALF samples may serve as diagnostic biomarkers of lung injury resulting from local or systemic inflammation, while mitochondrial material or other factors may serve as biomarkers of lung injury associated with microbial infection. However, unlike serum EVs that carry factors that can predict the development of ALI/ARDS, most EVs in BALF derive from damaged alveolar cells and thus carry RNA and protein biomarkers that reflect changes occurred after ALI/ARDS development.

In brief, EVs in BALF and serum derive from different cell types; however, EVs in BALF primarily derive from alveolar cells and neutrophils, while serum EVs reflect contributions from most systemic tissues (Table 2). Therefore, EVs detected in BALF samples may be a better choice for diagnosing ALI caused by external stimuli (e.g., respiratory pathogens and inhaled chemical irritants), since EVs released by alveolar macrophages, and perhaps other cell types, are likely to be enriched in the ASL. Conversely, serum EVs may be a better choice for diagnosing ALI induced by internal causes, since these EVs play an important role in the transfer of materials that cause endothelial dysfunction leading to ALI and are less likely to accumulate in ASL. In summary, both BALF and serum EVs can influence ALI/ARDS development, and partly reflect the etiology of ALI/ARDS, but only serum EVs predict the development and trend of the disease. Exploring gene or protein changes in serum/plasma EVs of patients with ALI/ARDS thus has strong potential to guide clinical decisions on early intervention measures to block the development of lung inflammation leading to ALI/ARDS.

**Table 2:** EVs in the diagnosis of ALI/ARDS

EVs source	Molecules	Prediction	References
Plasma	miR-34a, miR-15a and miR-27a	Shock	[106]
Serum	miR-126, miR-27a, miR-146a and miR-155	Severe community-acquired pneumonia-ARDS	[107]
Serum	miR-145	Sepsis	[108]
Alveolar macrophages	TNF and IL-10 protein	LPS-ALI	[109]
BALF	TNF, IL-1β/IL-6	LPS-ALI	[110]
BALF	Mitochondrial material	*Streptococcus pneumonia* infection	[111]

*EVs* extracellular vesicles, *ALI* acute lung injury, *ARDS* acute respiratory distress syndrome, *BALF* bronchoalveolar lavage fluid, *IL* interleukin, *TNF* tumor necrosis factor, *LPS* lipopolysaccharide

## Role of MSC-EVs in the treatment of ALI/ARDS

Numerous clinical studies on ALI/ARDS conducted within the past four decades have shown that a series of therapeutic strategies, including those based on surfactant, nitric oxide, anti-oxidant and immunotherapy, are ineffective [112]. MSCs have recently been shown to have promising therapeutic potential in ALI/ARDS through their action to modulate inflammation, reconstruct the alveolar epithelium and endothelium, and prevent pulmonary fibrosis [113]. MSCs can secrete soluble factors, such as growth factors, anti-inflammatory cytokines, and antimicrobial peptides, which can stabilize the alveolar barrier, enhance alveolar fluid clearance, and decrease infection; such factors have positive effects on the treatment of COVID-19 patients [114, 115], but MSC treatments are reported to increase the risk of iatrogenic tumor formation. MSC-mediated therapeutic effects are, in part, due to paracrine mechanisms mediated by EV release [114, 116]. For therapeutic applications, MSC-EVs have several advantages over MSCs, including low immunogenicity, long in vivo stability, high delivery efficiency, and low risk for inducing iatrogenic tumor formation [114, 117]. In this section, we will discuss the potential applications of EVs secreted by different stem cell types [e.g., bone marrow stem cells, adipose-derived stem cells, dental pulp stem cells (DPSCs), and human umbilical cord blood MSCs] in the regulation of lung tissue function to treat ALI/ARDS.

### Potential effects of MSC-EVs to attenuate ALI

ALI is typically characterized by alveolar edema, hemorrhage, and increased inflammatory cell and protein concentrations in ASL. MSC-EVs can also improve respiratory function in phosgene-induced ALI by reducing inflammation, inhibiting matrix metallopeptidase-9 (MMP-9) synthesis, and increasing the expression of surfactant protein-C [118]. More importantly, MSC-EV intervention prior to trauma-induced ALI can suppress inflammatory responses and improve oxidative stress injuries, ultimately inhibiting the development of ALI. This beneficial effect is largely mediated by EV transfer of miR-124-3P, which directly inhibits the expression of purinergic receptor P2X ligand-gated ion channel 7 (P2X7), a regulator of several inflammatory pathways that is overexpressed in rats with traumatic ALI [119].

Research also indicated that MSC-EVs can inhibit the activation of the pro-inflammatory TLR4/NF-κB signaling pathway to prevent or attenuate ALI. EVs from human umbilical cord MSCs can decrease serum and lung tissue levels of TNF-α, IL-1β and IL-6 in rats with burn-induced ALI via regulating the TLR4/NF-κB pathway by carrying miR-451 [120]. An MSC-EV-mediated inhibitory effect on the TLR4/NF-κB pathway was also observed in rats with ALI induced by intestinal ischemia–reperfusion, where MSC-EV treatment similarly reduced lung inflammation [121]. Together, these examples illustrate that MSC-EVs have great potential to reduce lung inflammation.

### Effects of MSC-EVs on the epithelium

Pulmonary epithelial cells, which play a key role in maintaining the stability of alveoli, are exposed to endogenous and external stimuli that can regulate their function. Multiple stimuli (e.g., sulfur mustard, hyperoxia and LPS) that induce apoptosis or autophagy or inhibit the proliferation of lung epithelial cells can induce epithelial barrier dysfunction and contribute to ALI [122–124]. MSC-EVs have been found to protect pulmonary epithelial cells from the harm of ALI. For example, the influenza virus can replicate in lung epithelial cells and induce apoptosis, but MSC-EVs can inhibit virus replication and the release of pro-inflammatory factors by transferring specific RNAs to lung epithelial cells that reduce lung inflammation [125].

Similarly, bone marrow MSC-EVs can inhibit epithelial cell apoptosis and restore their barrier function by up-regulating G protein-coupled receptor family C group 5 type A (GPRC5A) to promote the expression and re-localization of several junction proteins (e.g., E-cadherin, claudin-1, occludin, and ZO-1) [122]. MSC-EVs can also improve cell viability and prevent apoptosis by transferring miR-425 to epithelial cells to activate PI3K/Akt signaling to alleviate hyperoxia-induced ALI [123]. Further, the transfer of long non-coding RNAs (lncRNAs) by MSC-EVs can also play an important role in inhibition of lung epithelial cell apoptosis. For example, EV transfer of lncRNA-p21 was reported to suppress epithelial cell apoptosis and prevent ALI by down-regulating miR-181 and up-regulating sirtuin 1 (SIRT1) expression [124]. Endogenous levels of certain factors in MSC-EVs reduce lung epithelial cell apoptosis, but MSC-EVs can also be modified to overexpress beneficial molecules to improve their anti-apoptotic effects. For example, MSC-EVs modified to overexpress miR-30b-3p, which can inhibit LPS-induced AEC apoptosis, were found to increase the proliferation, and reduce the apoptosis of LPS-treated mouse lung epithelial cells [126].

Beyond their effects to attenuate apoptosis, MSC-EVs also have known activities to attenuate ALI by inducing autophagy, inhibiting inflammation, and restoring mitochondrial function. For example, human umbilical cord MSCs secrete EVs enriched with miR-377-3p, which can attenuate LPS-induced ALI by down-regulating mammalian target of rapamycin (mTOR) to inhibit the expression of inflammatory factors and induce autophagy, both in vivo and in vitro [127]. Another study found that DPSCs, particularly DPSCs modified to overexpress hepatocyte growth factor (HGF), had a stronger effect than umbilical cord MSCs to inhibit lung inflammation and fibrosis and improve the survival rate of a paraquat poisoning-induced ALI/ARDS mice model [128]. Similarly, MSC-EV treatment prevented AEC injury caused by mitochondrial gene damage in cigarette-exposed mice by transferring mitochondrial DNA with functional copies [129]. Thus, MSC-EVs may regulate lung AEC function and reduce lung injury through multiple mechanisms.

### Effects of MSC-EVs on endothelial cells

Approaches that target endothelial cells in the treatment of ALI are primarily designed to restore the normal function of the vascular endothelial barrier. MSC-EVs are reported to maintain the paracellular and transcellular permeability of lung microvascular endothelial cells following LPS treatment. This is accomplished by increasing the expression of the endothelial intercellular junction proteins VE-cadherin and occludin, decreasing endothelial apoptosis, and inducing endothelial cell proliferation [130, 131]. The effects of MSC-EVs on the pulmonary microvascular barrier are mediated by two mechanisms: 1) the transfer of angiopoietin-1 (Ang-1) mRNA, which has been reported to be an endothelial cell survival factor, and 2) the transfer of HGF, which leads to up-regulation of VE-cadherin and occludin [130, 131]. Adipose-derived stem cell-derived EVs (ADSC-EVs) have also been reported to alleviate ALI/ARDS by suppressing ventilator-induced injury to the pulmonary endothelial barrier by inhibiting the transient receptor potential vanilloid 4 (TRPV4)/Ca^2+^ pathway [132]. ADSC-EVs also attenuate histone-induced endothelial cell apoptosis via activation of the PI3K/Akt pathway [133]. In ALI caused by ischemia–reperfusion, MSC-EVs can also prevent pulmonary edema by attenuating the activation of immune cells and rebuilding the integrity of the endothelial cell barrier [134].

EVs derived from endothelial progenitor cells have also been reported to improve endothelial cell function. miR-126 was found to play a significant role in this process by down-regulating sprouty-related EVH1 domain-containing proteins 1 (SPRED1) expression to activate RAF/ERK signaling pathway, which enhanced the proliferation, migration, and capillary tube formation of recipient endothelial cells [135]. Further, the miR-126 content of endothelial progenitor cell exosomes has been reported to inhibit the expression of phosphoinositide-3-kinase regulatory subunit 2 (PI3KR2), the inflammatory alarmin high mobility group box protein 1 (HMGB1), and the permeability factor vascular endothelial growth factor α (VEGFα) to prevent endothelial dysfunction [6]. Researchers have also found that EVs secreted by human induced pluripotent stem cells (iPSCs) can effectively deliver miRNAs into human primary pulmonary microvascular endothelial cells to prevent ALI by silencing exosomes/siRNA gene, inhibiting intracellular adhesion molecule expression, and attenuating neutrophil adhesion [83]. MSC-EVs can thus restore the barrier function of pulmonary microvascular endothelial cells and improve ALI by decreasing apoptosis and inducing proliferation.

### Effects of MSC-EVs on alveolar macrophages

During lung injury, most monocytes recruited into the alveolar lumen differentiate into pro-inflammatory M1 macrophages that then amplify the pulmonary inflammatory response by secreting pro-inflammatory cytokines and chemokines. Thus, inhibiting the M1 polarization of these alveolar macrophages, or promoting anti-inflammatory M2 macrophage polarization represents an important therapeutic direction for alleviating ALI. The treatment of an in vitro LPS-induced ARDS model with bone marrow MSC-EVs inhibited LPS-induced M1 macrophage polarization by regulating the glycolysis pathway, leading to the accumulation of M2 macrophages associated with tissue repair [136]. Other in vivo and in vitro studies showed that MSC-EVs administered by inhalation were superior to caudal vein injection for promoting the polarization of macrophages to the M2 phenotype [137]. Apoptosis and autophagy of alveolar macrophages are also important factors contributing to LPS-related ALI. Bone marrow MSC-EVs have been reported to improve the survival of rats with LPS-induced ALI by improving alveolar macrophage autophagy and attenuating LPS-induced alveolar macrophage apoptosis [138], which may be accomplished through the transfer of miR-191 [139]. Additionally, EVs from human umbilical cord blood MSCs can protect against LPS-induced ALI by transferring miR-22-3p to attenuate LPS-induced expression of the frizzled class receptor 6 (FZD6). This ultimately inhibits inflammatory reactions and oxidative stress responses, increases alveolar macrophage proliferation, and reduces macrophage apoptosis [140]. Thus, MSC-EVs exhibit multiple effects on macrophages to protect against LPS-induced ALI.

### Effects of MSC-EVs on neutrophils

Neutrophil migration and lung infiltration and NET formation are important factors leading to the expansion of early-stage lung inflammation and lung injury. Thus, inhibiting neutrophil migration and NET formation could reduce the ALI/ARDS inflammatory response, and reports indicate that MSC-EVs possess this regulatory activity. For example, one study has reported that intratracheal infusion of MSC-EVs can reduce neutrophil influx and MIP-2 overexpression in the BALF of mice with endotoxin-induced ALI, partly through transfer of keratinocyte growth factor (KGF) mRNA to cells in injured alveoli [141]. Similarly, in a mouse pneumonia model, MSC-EVs were reported to significantly decrease BALF neutrophil influx and improve ALI via a miR-466-dependent process [142]. Another study has reported that MSC-EVs can transfer mitochondrial manganese superoxide dismutase to neutrophils to reduce oxidative stress, inflammation, and liver damage in a rat model of liver ischemia–reperfusion injury [143], and it is plausible that MSC-EVs can exhibit similar effects to reduce lung inflammation via a similar mechanism.

**Fig. 3:**
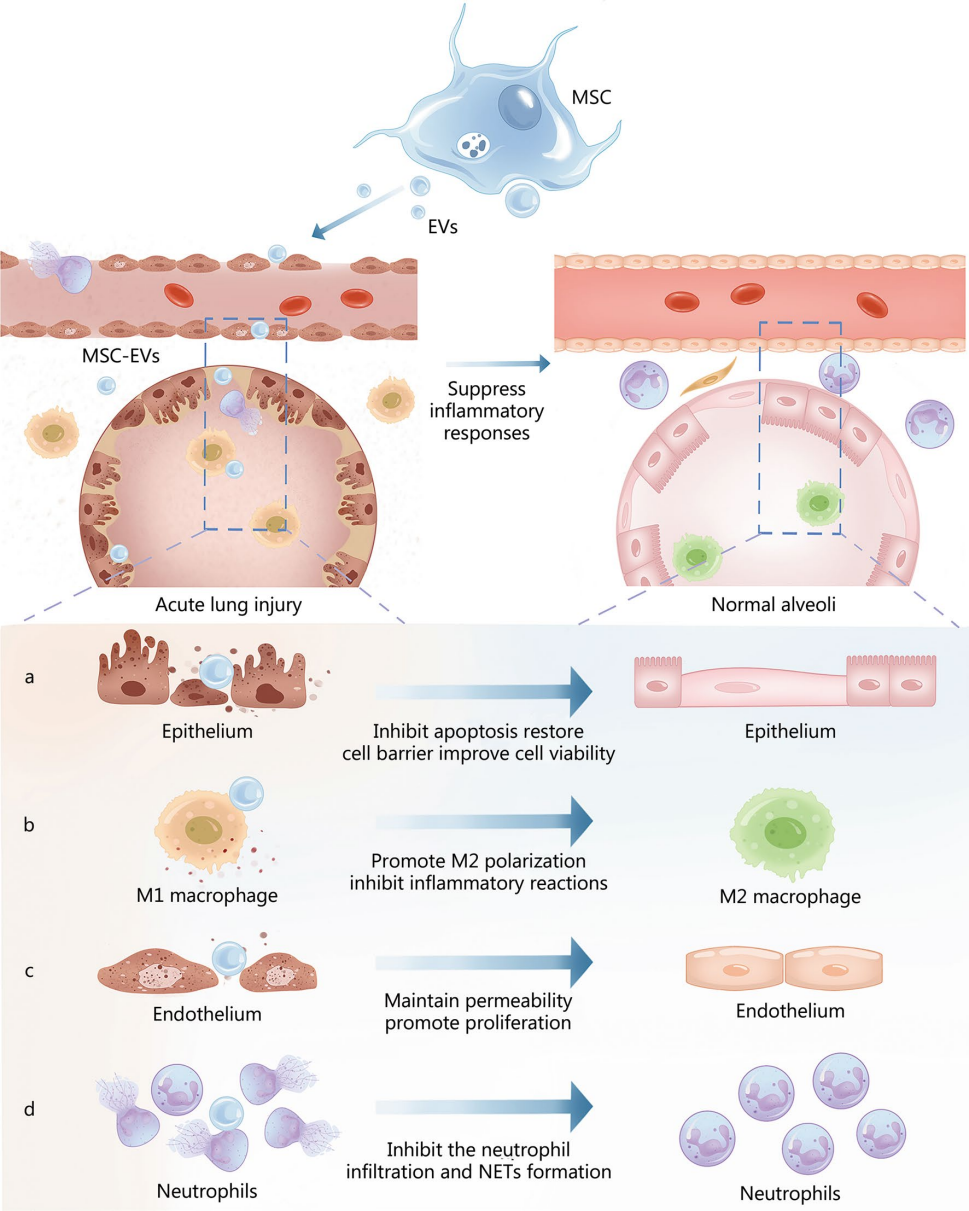
Mesenchymal stem cell-derived extracellular vesicles (MSC-EVs) can attenuate ALI/ARDS by suppressing inflammatory responses. **a** MSC-EVs inhibit epithelial cell apoptosis, restore epithelial cell barrier and improve cell viability. **b** MSC-EVs promote M2 polarization of alveolar macrophages and inhibit inflammatory reactions. **c** MSC-EVs maintain endothelial cell permeability and promote cell proliferation. **d** MSC-EVs inhibit the neutrophil infiltration and neutrophil extracellular traps (NETs) formation. ALI/ARDS acute lung injury/acute respiratory distress syndrome

ALI/ARDS can be induced by a variety of internal and external tissue insults, including direct and indirect mechanisms regulated by physical or chemical factors. As shown in Table 3 and Fig. 3, MSC-EV treatment can significantly reduce pulmonary inflammation by restoring the functions and structure of pulmonary epithelial cells and microvascular endothelial cells, polarizing anti-inflammatory macrophages, and inhibiting the neutrophil infiltration and NET formation. These positive effects are achieved by multiple effects, including inhibiting the occurrence of various pathological events (such as apoptosis), inducing proliferation, and improving autophagy. Notably, MSC-EVs can be engineered to carry specific cargoes, making them potentially potent vehicles to enhance the delivery of therapeutic molecules for ALI/ARDS treatment.

**Table 3:** MSC-EVs in the treatment of ALI/ARDS

ALI	EVs source	Molecules	Target	Effect	References
LPS	Endothelial progenitor cells-exosomes	miR-126	Endothelial cells	Enhanced endothelial cell proliferation, migration, and tube formation; reduced permeability and inflammation	[6]
LPS	Human iPSC-exosomes	Unknown	HMVECs	Inhibited ICAM-1 expression, and attenuated neutrophil adhesion	[83]
Phosgene	MSC-EVs	Unknown	Lung tissues	Modulated inflammation, inhibited MMP-9 synthesis and elevated SP-C levels	[118]
Traumatic	MSC-exosomes	miR-124-3P	Lung tissues	Inhibited P2X7 expression, suppressed inflammatory response and improved oxidative stress injury	[119]
Burn	HUCMSC-exosomes	miR-451	Lung tissues	Inhibited TLR4/NF-κB pathway/attenuated pulmonary inflammation	[120]
Sulfur mustard	Bone marrow MSC-exosomes	Unknown	Epithelial cells	Inhibited epithelial cell apoptosis and restored epithelial barrier function	[122]
Hyperoxia	Bone marrow MSC-exosomes	miR-425	Lung tissues/RLE-6TN cells	Up-regulated PI3K/Akt signaling, increased cell viability, and reduced apoptosis	[123]
Sepsis	MSC-exosomes	lncRNA-p21	Lung tissues/ epithelial cells	Down-regulated miR-181 and up-regulated SIRT1 expression/suppressed epithelial cell apoptosis and prevented ALI	[124]
LPS	MSC-EVs	Modified to exhibit miR-30b-3p	MLE-12 cells (mouse lung epithelial cells)	Increased cell proliferation and reduced apoptosis	[126]
LPS	HUCMSC-EVs	miR-377-3p	Lung tissues	Suppressed the inflammatory factors and induced autophagy, ameliorated ALI	[127]
Paraquat poisoning	DPSC-EVs	HGF	BESA-ZB	Inhibited the expression of pro-inflammatory factors and up-regulated the expression of anti-inflammatory factors	[128]
Cigarette	MSC-EVs	Mitochondrial DNA	BEAS-2B cells	Regulated early mitochondrial genes involved in the fission/fusion process	[129]
LPS	MSC-MVs	Ang-1 mRNA/HGF	Macrophages and HLMVECs	Maintained the integrity of microvascular endothelial cells and immunomodulatory properties of macrophages	[130, 131]
Ventilator	ADSC-EVs	Unknown	Endothelial cells	Inhibited the TRPV4/Ca^2+^ pathway/suppressed pulmonary endothelial injury and inflammatory responses	[132]
Histone	MSC-exosomes	miR-126	HUVECs	Activated the PI3K/Akt pathway/attenuated endothelial cell apoptosis	[133]
Ischemia–reperfusion	MSC-EVs	Unknown	Endothelial cells	Attenuated the activation of immune cells and restored the integrity of the endothelial cell barrier	[134]
LPS	Endothelial progenitor cell-EVs	miR-126	Endothelial cells	Down-regulated SPRED1 expression to activate RAF/ERK signaling, enhanced proliferation, migration and capillary tube formation	[135]
LPS	MSC-exosomes	Unknown	Macrophages	Inhibited cellular glycolysis and M1 macrophage polarization	[136]
LPS	MSC-EVs	Unknown	Alveolar macrophages	Improved autophagy/attenuated viability loss and apoptosis	[139]
LPS	HUCMSC-exosomes	miRNA-22-3p	Alveolar macrophages	Inhibited inflammatory reaction and oxidative stress response, increased alveolar macrophage proliferation, and reduced macrophage apoptosis	[140]
*Escherichia coli* endotoxin	MSC-MVs	KGF mRNA	Neutrophils	Reduced the influx of neutrophils and MIP-2 levels in BALF	[141]
MDR-PA pneumonia	MSC-EVs	miR-466	Neutrophils	Decreased influx of BALF neutrophils, proinflammatory factor levels and proteins	[142]

*ADSC* adipose-derived stem cell, *ALI* acute lung injury, *ARDS* acute respiratory distress syndrome, *BEAS-2B* human bronchial epithelial cells, *BALF* bronchoalveolar lavage fluid, *DPSC* dental pulp stem cell, *HGF* hepatocyte growth factor, *HLMVECs* human lung microvascular endothelial cells, *HMVECs* human primary pulmonary microvascular endothelial cells, *HUCMSC* human umbilical cord mesenchymal stem cells, *iPSC* induced pluripotent stem cell, *ICAM-1* intercellular cell adhesion molecule-1, *KGF* keratinocyte growth factor, *LPS* lipopolysaccharide, *MDR-PA* multidrug-resistant *Pseudomonas aeruginosa*, *MIP-2* macrophage inflammatory protein-2, *MMP-9* matrix metallopeptidase-9, *MSC-EVs* mesenchymal stem cell-derived extracellular vesicles, *P2X7* P2X ligand-gated ion channel 7, *SP-C surfactant protein-C, SPRED1* sprouty-related EVH1 domain-containing proteins 1

## Conclusions and outstanding questions

EV-based therapies have significant potential to attenuate lung injury to prevent ALI/ARDS, but there are a number of critical parameters that need to be standardized before such therapies can be tested in preclinical studies and clinical trials. First, because EV cargoes can change in response to stimuli encountered by their parent cells, the effects of MSC culture and pre-treatment conditions on EV function must be carefully optimized and standardized to ensure reliable therapeutic outcomes. For instance, EVs isolated from MSCs cultured in a differentiation medium that induced the expression of neurotrophic and immunoregulatory factors were found to have enhanced ability to attenuate ARDS phenotypes in a LPS-induced mouse model of pulmonary inflammation and tissue injury [144]. Second, the development and production of EV therapeutics require the use of large-scale, standardized EV isolation procedures that carefully characterize and quantify isolated EV samples to ensure their reproducible performance [114]. Large-scale EV production is necessary for ALI/ARDS therapeutics as roughly 5- to 10-fold more MSCs are required to produce an MSC-EV dose that matches the therapeutic effect of direct treatment with MSCs [114]. New, reproducible and large-scale EV isolation techniques are needed to address this demand. Future studies are also required to evaluate the optimal administration method and doses for EV therapeutics, as well as their risks and clinical efficacy in preventing or attenuating ALI/ARDS. Strict culture requirements can also complicate the production of undifferentiated MSCs at scale. Recently, however, engineered fibroblast-secreted EVs [activated specialized tissue effector extracellular vesicles (ASTEX)] have been shown to attenuate lung inflammation by inhibiting the production of pro-inflammatory factor IL-6 and promoting the expression of anti-inflammatory factor IL-10 in primary macrophages [145]. ASTEX have shown good safety in a long-term study of ALI mice, and the cells that produce ASTEX can be readily cultured and modified in vitro to permit their large-scale production, making it a very promising EV-based treatment method for ALI/ARDS.

In summary, the effects of EVs s on cell dysfunction in pulmonary epithelial, endothelia cells, alveolar macrophages, and neutrophils are associated with the development of lung inflammation and injury that can result in ALI/ARDS. Activated or damaged lung cells can secrete EVs that carry factors associated with their injury phenotypes in the ASL or peripheral circulation, which can be used as biomarkers to diagnose and predict the progression of lung injuries using BALF, plasma, or serum samples. Moreover, such EV markers may also represent specific targets for therapeutic intervention to attenuate lung injury and prevent ALI/ARDS development. MSC-EV is shown to be a promising agent to reduce or even cure ALI/ARDS through multiple mechanisms that attenuate the inflammation, dysregulation, and apoptotic responses mediated by or affecting different pulmonary cell types.

## Data Availability

Not applicable.

## Abbreviations

ADSC: Adipose-derived stem cell
AECs: Alveolar epithelial cells
AEC-Is: Type I alveolar epithelial cells
AEC-IIs: Type II alveolar epithelial cells
AECC: American-European consensus conference
ALI: Acute lung injury
Ang-1: Angiopoietin-1
ARDS: Acute respiratory distress syndrome
ASL: Airway surface liquid
BALF: Bronchoalveolar lavage fluid
BEAS-2B: Human bronchial epithelial cells
COPD: Chronic obstructive pulmonary disease
DPSCs: Dental pulp stem cells
EVs: Extracellular vesicles
FZD6: Frizzled class receptor 6
GPRC5A: G protein-coupled receptor family C group 5 type A
HGF: Hepatocyte growth factor
HMGB1: High mobility group box protein 1
HPMECs: Human pulmonary microvascular endothelial cells
HLMVECs: Human lung microvascular endothelial cells
iPSCs: Induced pluripotent stem cells
ICAM-1: Intercellular adhesion molecule-1
ICAM-2: Intercellular adhesion molecule-2
IL: Interleukin
KGF: Keratinocyte growth factor
lncRNAs: Long noncoding RNAs
LPS: Lipopolysaccharide
MIP-2: Macrophage inflammatory protein-2
MMP-9: Matrix metallopeptidase-9
MSCs: Mesenchymal stem cells
mTOR: Mammalian target of rapamycin
MVs: Macrovesicles
NETs: Neutrophil extracellular traps
NLRP3: NLR family pyrin domain containing 3
OXLDL: Oxidized low-density lipoprotein
P2X7: P2X ligand-gated ion channel 7
PIK3R2: Phosphoinositide-3-kinase regulatory subunit 2
ROS: Reactive oxygen species
S100A9: S100 calcium binding protein A9
SERP1: Stress-associated endoplasmic reticulum protein 1
SHIP1: SH2 domain-containing inositol phosphatese1
SIRT1: Sirtuin 1
SOCS1: Suppressor of cytokine signaling 1
SPRED1: Sprouty-related EVH1 domain-containing proteins 1
TNF: Tumor necrosis factor
TRPV4: Transient receptor potential vanilloid 4
VEGFα: Vascular endothelial growth factor α
ZO-1: Zonula occludens-1
